# Overexpression of *GmCSY3* Enhances Soybean Tolerance to Excess Iron and Aluminum

**DOI:** 10.3390/biology15010105

**Published:** 2026-01-05

**Authors:** Zhuo Liu, Hongqiu Lv, Liying Yang, Yu Wang, Xinqi Zhu, Menghan Chang, Wenwei Liang, Shanshan Wang, Ying Yang, Yining Pan, Changhong Guo, Yingdong Bi, Donglin Guo

**Affiliations:** 1 Heilongjiang Provincial Key Laboratory of Molecular Cell Genetics and Genetic Breeding, College of Life Science and Technology, Harbin Normal University, Harbin 150080, China; lz13329317581@163.com (Z.L.); 13359935995@163.com (H.L.); 17703647519@163.com (L.Y.); wangyu12112@163.com (Y.W.); z1273424841@163.com (X.Z.); cmh6650@163.com (M.C.); 13613626395@163.com (S.W.); 18346316087@163.com (Y.Y.); panyining2023@163.com (Y.P.); kaku3008@hrbnu.edu.cn (C.G.); 2 Institute of Crops Tillage and Cultivation, Heilongjiang Academy of Agricultural Sciences, Harbin 150086, China; liangwenwei5@163.com

**Keywords:** *GmCSY3*, citrate synthase, soybean, Fe, Al, ROS

## Abstract

Background: Plant citrate synthase (CSY) is involved in the iron deficiency (−Fe) response and aluminum (Al) detoxification. However, knowledge of CSY function in responding to excess iron (+Fe) or Al stress (+Al) is still limited. Methods: The CDS and promoter of *GmCSY3* were isolated from soybean and bioinformatically analyzed. The *GmCSY3* expression was detected by qRT-PCR and GUS assay. The growth of *GmCSY3* recombinant yeast under +Fe or +Al was detected. The phenotype, CSY activity, citric acid concentration, chlorophyll content, MDA, H_2_O_2_, O_2_^−^ contents, GST, CAT, SOD, and POD activities were examined in *GmCSY3* overexpressed and RNAi-suppressed soybean chimeras under +Fe or +Al. Perls and Hematoxylin stained the roots, and the FCR activity was determined. Results: *GmCSY3* was induced by +Fe or +Al, but not by −Fe. *GmCSY3* enhanced yeast’s acid production and resistance to +Fe or +Al. *GmCSY3* overexpression in soybean significantly enhanced CSY activity, promoted growth, alleviated oxidative damage caused by +Fe or +Al, with less free Fe^3+^ and Al^3+^, and reduced FCR activity, while *GmCSY3* RNAi-suppressed showed the opposite effect. Conclusions: *GmCSY3* promotes the process of citrate synthesis, chelates Fe^3+^ and Al^3+^, alleviates oxidative damage caused by +Fe or +Al, and modulates iron absorption in plants.

## Simple Summary

Plants growing in acidic soil are prone to poisoning from excessive Fe or Al, which can lead to growth damage. We obtained the *GmCSY3* gene of soybean, which is involved in the synthesis of citric acid. The expression of *GmCSY3* became more active when soybean roots were exposed to high levels of Fe or Al. Experiments demonstrated that increasing the expression level of *GmCSY3* enhanced the activity of citrate synthase, which subsequently binds to excess Fe and Al, resulting in reduced damage in plants exposed to excessive Fe and Al. Conversely, reducing the expression level of *GmCSY3* in plants reduced citrate synthase activity and caused greater harm to the plants. Understanding the process involved in this *GmCSY3* gene can help develop more resistant crops or new application supplements to better cope with acidic or contaminated soil, contributing to better food production.

## 1. Introduction

Aluminum (Al) and iron (Fe) are abundant elements in the Earth’s crust. Due to their transition element characteristics, both metals are more easily absorbed by plants in low-pH soils. Soil acidity, affecting more than 40% of the world’s potentially arable land, severely constrains global agricultural productivity [1,2,3]. Al^3+^ rapidly inhibits root elongation by disrupting apical cellular processes and membrane function, impairing water and nutrient uptake [4,5]. Fe^2+^ accumulation triggers oxidative stress via the Fenton reaction, leading to chlorosis and necrosis, which negatively affects plant growth and development [6,7].

Citric acid (CA), a central tricarboxylic acid (TCA) cycle intermediate, fulfills fundamental roles in plants’ core metabolism [8,9,10], development [11,12], and stress response to abiotic stress [13,14,15,16], nutritional element deficiency [17,18], and metal stress [19,20,21]. CA acts as a pivotal chelator for storage and transport of Al^3+^ [22,23,24,25,26] and Fe^2+^ and may function as a signaling molecule reflecting cellular Fe status [27,28,29,30]. Citric acid synthase (CSY), as the rate-limiting enzyme for citrate synthesis, catalyzes the condensation of oxaloacetate and acetyl-CoA, serving as the primary entry point into the TCA cycle and thus regulating cellular citrate flux [31]. *CSY* genes were isolated and characterized in a few plants [32,33,34,35], etc. *CSY* gene expression is spatiotemporally dynamic, regulated intrinsically by species, genotype, tissue ontogeny, and metabolic demand, and extrinsically by abiotic stressors [36,37]. However, the functional contribution of *CSY* genes to modulating citrate pools, which are essential for concurrent Al tolerance and Fe homeostasis, remains largely unexplored.

CA is the leading intercellular and intracellular Fe chelating agent, and Fe is mainly present in the xylem in the form of Fe^3+^-citrate for root to stem transporting [38,39]. As an Fe reservoir, ectoplasmic Fe can be activated and reused when plants face an Fe-deficient environment. *MATE* family members mediate the expulsion of citric acid from xylem periductal parenchyma cells to xylem and the transport of Fe^3+^-citric acid chelates [40,41]. Fe stress strongly induced the expression of *MxCS1*, *MxCS3*, and *MdCS1* genes in plants. Overexpression of *MxCS1*, *MxCS3,* and *MdCS1* promoted the synthesis of citrate synthase, increased the concentration of CA, and improved the tolerance of transgenic Arabidopsis to Fe stress, suggesting that the *CSY* genes may play a regulatory role in plant response to Fe stress [42,43,44].

In order to cope with Al toxicity, plants have evolved various anti-Al toxicity response mechanisms. For example, by secreting organic acids such as malic acid and citric acid into the rhizosphere or vacuole to form stable organic acid Al chimeras, thereby reducing Al uptake [45,46,47]. Although there has been debate as to whether enhanced organic acid anabolism in vivo is associated with Al-induced organic acid secretion [48], some studies have shown that increased citric acid secretion is associated with changes in *CS* gene expression and rice mitochondrial gene *OsCS1* [49], along with the increase in citric acid content and enzyme activity [5,50]. Researchers overexpressed some organic acid synthase genes, including citrate synthase, malate dehydrogenase, pyruvate phosphate dikinase, and Phosphoenolpyruvate carboxylase, which increased Al tolerance [51,52,53,54,55]. However, researchers seem to pay more attention to its transporter proteins, for example, the ALMT, MATE ALS, STAR, and Nrat transporters, which are thought to be associated with Al toxicity tolerance mechanisms [56,57,58,59,60]. Considering that the types of organic acids released by plants under Al^3+^ stress are limited and specific, some researchers believe that the existence of specific organic acid transport channels or carriers on the cell membrane may be the main factor limiting the secretion of organic acids and improving the Al tolerance. In summary, the mechanism of *CS* in plant Al resistance is complex and worth studying.

In recent years, some genetic engineering studies on citrate synthetase genes in plants have been successfully carried out. Overexpression of mitochondrial citrate synthase in carrot cells increased the growth rate of Arabidopsis in Al phosphate medium [61]. Overexpressed rice *OsCS* [33], pomelo *CjCS* [49], rye and two-spike malt [36], and *Rhododendron micranthum Turcz*. The *RmCS* gene [35] increased the level of citrate in the recipient plant and improved tolerance to Al. The improvement in plant Al resistance by citric acid synthase includes regulating energy metabolism, improving photosynthetic efficiency, inducing the antioxidant defense system, reducing ROS, and other ways [13]. These findings suggest that overexpression of suitable *CSY* genes may be a valuable tool for achieving Al tolerance in plants. Further, overexpression of *GmCS*, *MxCS1* [62], *MxCS3* [43], or *MdCS1* [42] enhances Fe chelation and tolerance to iron deficiency. Some genes, such as *NPF*, *GmYSL7*, and *OsIET1*, were revealed to be involved in iron homeostasis in Arabidopsis, soybean, and Rice, whose functional abnormalities impair plant development [63,64,65]. It has been demonstrated that the GmALS3 protein in soybeans could serve as a biomarker for aluminum toxicity [66]; The phosphorylation state of the ALR1 protein in Arabidopsis affected the plant’s perception of Al [67]; GmSTOP1-3 enhanced Al tolerance by promoting flavonoid biosynthesis [68].

Despite these advances, critical knowledge gaps remain: the molecular basis for discordant CS protein levels and citrate accumulation, the direct link between intracellular citrate metabolism and Al-induced secretion, and the integration of CS regulatory networks across Al/Fe stresses are poorly understood. Al toxicity first damages plant roots, inhibiting root tip division and elongation, causing morphological abnormalities, and interfering with nutrient uptake and water transport. It then impairs photosynthetic pigments and enzyme activities, triggers oxidative stress, and ultimately leads to stunted plant growth and even death in severe cases [5,45,46,47]. Elucidating these mechanisms will not only deepen our understanding of plant adaptive metabolism but also enable rational engineering of CS-mediated pathways to enhance crop resilience to acidic soils and nutrient-limited environments.

Soybean (*Glycine max* L.) is one of the most important dicotyledonous crops and a good source of protein, fat, and dietary fiber, which can be used to produce a variety of products. Al toxicity affects the growth and yield of soybeans [69,70,71,72,73]. Fe deficiency is one of the major nutritional disorders affecting soybean production [74,75]. Fe chelate reductase (FCR) not only serves as an important enzyme in Fe transport, but also acts as an indicator of iron status in plants [75]. Although citrate synthase has emerged as a hub gene in abiotic stress responses (e.g., salt tolerance [76]), the specific roles of GmCS isoforms in mediating adaptive responses to Al toxicity and Fe homeostasis remain poorly defined in soybean, limiting targeted genetic improvement for acidic soil resilience. Here, through comprehensive physiological, biochemical, and histochemical analyses of *GmCSY3* overexpression and RNA interference transgenic lines, we demonstrate that GmCSY3-derived citrate alleviates Fe toxicity by inhibiting FCR activity and reducing Fe accumulation while mitigating Al toxicity via enhanced citrate excretion and internal chelation processes that lower root Al burden and preserve root function. Collectively, our findings establish *GmCSY3* as a central modulator of soybean’s dual tolerance to Al and Fe toxicities, highlighting its potential as a target for improving acidic soil adaptation. This work advances the understanding of citrate-mediated metal tolerance mechanisms and provides a foundation for sustainable soybean improvement in marginal acidic soils.

## 2. Materials and Methods

### 2.1. Plant Material and Growth Conditions

Heinong51 (HN51) soybean seeds were hydroponically cultivated with 1/2 Hoagland solution. The young soybean seedlings were used for DNA and RNA extraction. For qRT-PCR analysis, the roots, stems, and leaves were sampled at the three-leaf stage from HN51 soybean seedlings hydroponically cultivated, and the flowers, pods, and seeds were sampled from HN51 soybean seedlings planted in the experimental field. Germinated Williams 82 soybean young seedlings were used to induce hairy roots. The soybean chimeras were hydroponically cultivated.

### 2.2. Cloning of the GmCSY3 Gene and Its Promoter

Total RNA was extracted from HN51 seedlings using Total RNA Kit II (Omega BioTek, Norcross, GA, USA) and reverse transcribed to cDNA using ReverTra Ace™ qPCR RT Master MIX (TOYOBO, Osaka, Japan). The coding sequence of *GmCSY3* was amplified from cDNA with primers shown in Supplementary Table S1. The total DNA of HN51 was extracted using the E.Z.N.A.^®®^ SP Plant DNA Kit (OMGAE, New York, NY, USA). The promoter of *GmCSY3* was amplified from DNA with primers shown in Supplementary Table S1. After purification, PCR products were sequenced (Sangon Biotechnology Co., Ltd., Shanghai, China) and analyzed using BLAST (https://www.ncbi.nlm.nih.gov/orffinder/, accessed on 4 November 2023).

### 2.3. Bioinformatic Analysis of the GmCSY3 Gene and Its Promoter

A total of 59 CSY protein sequences of 56 plant species were downloaded from NCBI (https://www.ncbi.nlm.nih.gov/, accessed on 28 November 2023) and Phytozome (https://phytozome-next.jgi.doe.gov/info/Gmax_Wm82_a2_v1/, accessed on 28 November 2023). The phylogenetic tree was constructed using the Neighbor Joining (NJ) method in MEGA 11. The Hidden Markov Model (HMM) for the analysis of 11 *GmCSY* gene families in soybean, with CSY (PF09611) downloaded from the Pfam database, was constructed using the Neighbor Joining (NJ) method in MEGA 11. The phylogenetic tree classification was determined based on both evolutionary distance and motif analysis. The conservation of GmCSY protein motifs was analyzed using MEME 5.5.4. The gene replication relationship is processed through Putty 0.81.0.0 for program execution, with TBtools-II providing visualization. The structural domain of the GmCSY3 protein was predicted using NCBI protein Blast, and the tertiary structure of the GmCSY3 protein was predicted using SWISS-MODEL (https://swissmodel.expasy.org/, accessed on 23 December 2023). The amino acid sequence alignment of *Glycine max*, *Glycine soja*, *Vigna angularis*, Arabidopsis, *Oryza sativa*, and *Citrus clementina* was performed using DNAMAN 9.0. A 2000 bp upstream fragment of the *GmCSY* gene was employed as the promoter sequence, and PlantCARE (http://bioinformatics.psb.ugent.be/webtools/plantcare/html/, accessed on 18 April 2023) was used to analyze cis-acting elements in the promoter region. The analysis result of the *GmCSY3* promoter is shown in Supplementary Table S2. The promoter element results were categorized and statistically analyzed according to growth and development, biotic/abiotic stress, light response elements, and hormone response. The final results were visualized using TBtools-II.

### 2.4. Expression Analysis of GmCSY3

The roots, stems, leaves, flowers, pods, and seeds of HN51 soybean were frozen in liquid nitrogen and stored at −80 °C to detect the tissue expression of *GmCSY3* with specificity primers shown in Supplementary Table S1. When the hydroponically cultivated HN51 plants reached the three-leaf stage, the seedlings were transferred into 1/2 Hoagland solution without Fe (Fe deficiency, −Fe) or with 150 μM FeSO_4_ (excess Fe, +Fe) for 7 d, respectively. For Al stress (+Al), HN51 soybean plants were cultured in 1/2 Hoagland solution containing 300 μM AlCl_3_ for 24 h in a solution of CaCl_2_ (pH = 4.5). The 1/2 Hoagland solution cultivation was used as the control (CK). The underground and above-ground parts of the treated seedlings were, respectively, sampled and stored at −80 °C for RNA extraction. qRT-PCR was performed on the isolated RNA using SYBR^®^ Premix Ex Taq™ II (TAKARA, Otsu, Japan) by a CFX96 real-time PCR detection system (Bio-Rad, Hercules, CA, USA). The reaction system was as described above, and the relative gene expression was calculated using the ΔΔCt method.

### 2.5. GUS Assay

The soybean was infected with pBI121-*GmCSY3*pro::GUS and cultivated for 30 d. The hairy roots were cultivated in 1/2 Hoagland nutrient solution. The soybean hairy roots were treated with 1/2 Hoagland nutrient solution containing 0 µM Fe-EDTA, 50 µM Fe-EDTA, and 150 µM Fe-EDTA, respectively, for 7 d. After pre-culturing in 0.5 mM CaCl_2_ (pH = 4.5) for 12 h, the roots were exposed to 300 μM AlCl_3_ in CaCl_2_ solution (pH = 4.5) for 24 h. GUS staining was conducted following the method described previously [77]. The stained tissues and temporary mounts of root cross-sections were photographed using a stereomicroscope (EZ4-HD LEICA, Wetzlar, Germany) coupled with a color charge-coupled device (CCD) camera (Zeiss, Oberkochen, Germany). The GUS enzyme activity was detected using a plant β-glucuronidase (GUS) ELISA kit (KQ13690, Keqiao Biology, Xiamen, China). The OD values of each sample were measured with FlexA-200 enzyme marker (Ferdi Bio, Hangzhou, China) at 450 nm, with the activity value of the standard product as the horizontal coordinate and the corresponding OD value as the longitudinal coordinate. The linear regression curve was drawn, and the linear regression equation was calculated.

### 2.6. Stress Tolerance Test of GmCSY3 Recombinant Yeast

The *GmCSY3* gene fragment was connected to the pYES2 vector fragment using *Hind III* and *Xba Ι*, and the product was transformed into the *Saccharomyces cerevisiae* (INVSC1) with pYES2 as the control. The single colonies of pYES2 and pYES2-*GmCSY3* transformed yeast were extracted and inoculated in SC-U liquid medium for 24 h at 30 °C. Measure the pH value using a pH meter (PHS-3C, Lei Ci, Shanghai, China). The solution was collected, centrifuged at 4000 rpm for 1 min. The pH value of the supernatant was measured using the aforementioned method. 10 mL SC-U (+glucose) was used to induce the medium to resuspend yeasts to OD_600_ = 0.4. After induction culture at 30 °C for 36 h, the cultivate solution was collected, centrifuged at 4000 rpm for 1 min, the supernatant was discarded, and the yeast were suspended in sterilized water to OD_600_ = 2.0; The solution was diluted to 10^−1^, 10^−2^, 10^−3^, and 10^−4^ times successively, and 3 μL diluted solution was inoculated on SC-U (+glucose) solid medium and cultured at 30 °C. The treatment concentration was 50, 75, 100 mM for FeSO_4_ and 30, 40, 50 mM for AlCl_3_, with 0 mM as the control (CK). The growth state of the yeast colony was observed.

### 2.7. Vector Construction and Plant Genetic Transformation

The *GmCSY3* CDS was connected with the pBI121 vector to obtain pBI121-*GmCSY3*. The design of the RNAi fragment was performed by obtaining the homologous genes of *GmCSY3* using NCBI, conducting sequence alignment with DNAMAN, and selecting the sequence with the highest specificity from the conserved sequences. This sequence is located between 500 bp and 840 bp of *GmCSY3*, with a length of 341 bp. The *GmCSY3* RNAi gene fragment was ligated with the pZH01 vector to obtain pZH01-*GmCSY3*. The product was transformed into *Escherichia coli* DH5α and then transformed into *Agrobacterium tumefaciens* K599. The *Agrobacterium tumefaciens*-mediated ligation method was used to infiltrate soybeans to induce hairy roots and obtain the *GmCSY3* overexpression, RNAi-interfering, and control soybean chimeras. The qRT-PCR analysis was performed on hairy roots of soybean chimeras.

### 2.8. Citrate Synthase Activity and Citric Acid Concentration Detection

The soybean chimeras with *GmCSY3* overexpression and RNAi were cultured for about 10 d, and the citrate synthase activity of transgenic soybean hairy roots was determined by spectrophotometry using the citrate synthase kit (M0702C, MICHY BIOLOGY, Baoding, China). The concentrations of citric acid (CA) were determined using the Citric Acid Assay Kit from Mengxi Biotechnology (Catalog No.: 0703B; Format: 48 assays, Baoding, China), following the manufacturer’s protocol with minor modifications.

### 2.9. Physiological Index Detection

The soybean chimeras were stressed by Fe and Al, as described in 2.4. The content of chlorophyll was determined by the 80% acetone, and the light absorption values of the solution were tested at 663 nm and 645 nm [78]. Root length was measured using a ruler. The NBT staining solution was prepared using a 50 mM PBS buffer to a concentration of 0.5 mg/mL (pH 7.8) and used immediately. The staining solution was stored in the dark at 28 °C for 6 h, then decolorized with 90% ethanol in a 70 °C water bath. The leaf image scanning was performed using the root system analysis system (MICROTEK, Hangzhou, China). The content of malondialdehyde (MDA) was determined according to Rajinder with slight modifications [79]. The content of hydrogen peroxide (H_2_O_2_) and superoxide anion (O_2_^−^) was determined according to Veljovic-Jovanovic et al. [80]. The glutathione S-transferase (GST) activity was measured using the GST Assay Kit (M0305B, Mengxi Bio, Baoding, China). The activities of catalase (CAT), superoxide dismutase (SOD, WST-1 method), and peroxidase (POD) were detected using their corresponding assay kits (CAT: BC0205; SOD: BC5165; POD: BC0095; all from Solarbio, Beijing, China), respectively. Ferric chelate reductase (FCR) activity was determined using a specific assay kit (G0147F, Geruisi, Suzhou, China).

### 2.10. Histochemical Staining

For Perls staining, an equal-volume mixture of 4% (*v*/*v*) hydrochloric acid and 4% (*w*/*v*) potassium ferricyanide was prepared as the staining solution. Transgenic soybean hairy roots were vacuum-infiltrated with the solution and stained at 27 °C for 2–3 h. The sample was observed under a stereo microscope. For Hematoxylin staining, the hairy root tips of soybean were soaked in distilled water for 15 min, the residual Al on the surface of the root tips was washed off, and the stained hairy root was soaked in 0.l% hemoxylin dye solution (containing 0.01% KIO_3_) for 20 min, and then the dyed hairy root was soaked in distilled water for 10 min. The sample was observed under a stereo microscope. Quantitative analysis of staining results was performed using ImageJ 13.0.6 to obtain the final mean intensity per unit.

### 2.11. Statistical Analysis

Each treatment group included three biological replicates. To minimize measurement errors, three technical replicates were performed for each biological replicate. The technical replicate data of each sample were averaged prior to analysis, and statistical significance analysis was based on the data from the three biological replicates. IBM SPSS Statistics 27 statistical software was used for statistical analysis, with * indicating *p* < 0.05 and ** indicating *p* < 0.01. Lowercase letters indicate *p* < 0.05, and Capital letters indicate *p* < 0.01. Excel 2019 was used to organize and draw the data.

## 3. Results

### 3.1. Cloning and Bioinformatics Analysis of GmCSY3

**Figure 1:**
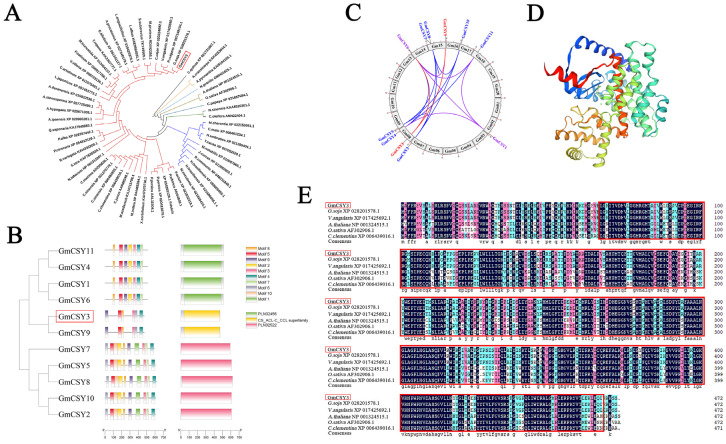
Bioinformatics analysis of the *GmCSY3* gene. (**A**) Phylogenetic tree of the 59 CSY proteins from 56 species. *G. max*, *G. soja*, *V. angularis*, *P. vulgaris*, *A. precatorius*, *A. hypogaea*, *C. cajan*, *M. pruriens*, *L. japonicus*, *G. bilobum*, *L. albus*, *L. angustifolius*, *A. duranensis*, *A. stenosperma*, *C. arietinum*, *T. repens*, *M. truncatula*, *V. villosa*, *P. sativum*, *P. cineraria*, *P. alba*, *B. variegata*, *A. pycnantha*, *A. crassicarpa*, *A. ipaensis*, *S. tora*, *X. sorbifolium*, *M. esculenta*, *H. brasiliensis*, *N. gracilis*, *J. curcas*, *C. sinensis*, *C. clementina*, *M. indica*, *C. maxima*, *C. junos*, *P. avium*, *M. notabilis*, *N. sinensis*, *R. communis*, *P. sibirica*, *M. azedarach*, *M. charantia*, *P. dulcis*, *C. papaya*, *P. persica*, *C. melo*, *H. umbratica*, *C. quinoa*, *T. cacao*, *C. oleifera*, *A. thaliana*, *O. sativa*, *N. tabacum*, *S. suberectus*, *Q. saponaria.* (**B**) Phylogenetic analysis, CDD, and motif analysis of CSY proteins in soybean. (**C**) Duplication relationships of CSY genes in soybean. (**D**) Predicted tertiary structure of GmCSY3 protein. Blue and red represent α-helices; green and yellow represent β-sheets; orange represents random coils or β-turns. (**E**) The sequence alignment of GmCSY3 and CSY proteins from *Glycine soja*, *Vigna angularis*, *Arabidopsis thaliana*, *Oryza sativa*, and *Citrus clementina.* The same color indicates similar physicochemical properties. The conserved structural domain is framed in red.

The *GmCSY3* CDS was obtained by PCR from the cDNA of *Glycine max* Heinong51 (Supplementary Figure S1). Evolutionary analysis indicated that GmCSY3 (XM_003531407.5) has a close genetic relationship with other CSY proteins. GmCSY3 and CSY protein (G. soja_XP028201578.1) of *Glycine soja* are distributed in the same branch, with an especially close genetic relationship (Figure 1A). There is a total of 11 *CSY* genes in the genome of *Glycine max*. The structural characteristics of GmCSY3 and GmCSY9 are the most similar, significantly differ from other GmCSY proteins of the other two subgroups (Figure 1B). *GmCSY3* is located on chromosome 8 of the soybean genome and has a gene replication relationship with *GmCSY9* and *GmCSY6* (Figure 1C). The CDS of *GmCSY3* is 1419 bp, encoding a hydrophobic citric synthase. GmCSY3 protein consists of α helix (55.72%), random coil (28.82%), and extended chain (8.26%) (Figure 1D). The conserved amino acids of GmCSY3 are similar to those of CSY proteins in *Glycine max*, *Glycine soja*, *Vigna angularis*, *Arabidopsis thaliana*, *Oryza sativa*, and *Citrus clementina*, containing a citric acl-CoA lyase superfamily domain (CS_ACL-C_CCL superfamily domain) (Figure 1E).

### 3.2. Promoter Element Analysis and Expression Characteristics of GmCSY3

**Figure 2:**
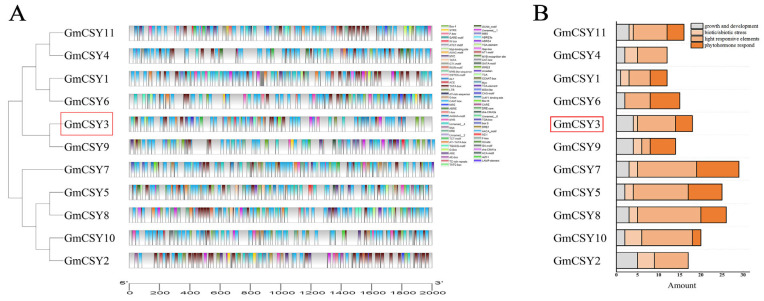
Bioinformatics analysis of the promoter of *CSY* genes in soybean. (**A**) Cis-elements. (**B**) Classification of the cis-elements. *GmCSY3* is framed in red to highlight.

**Figure 3:**
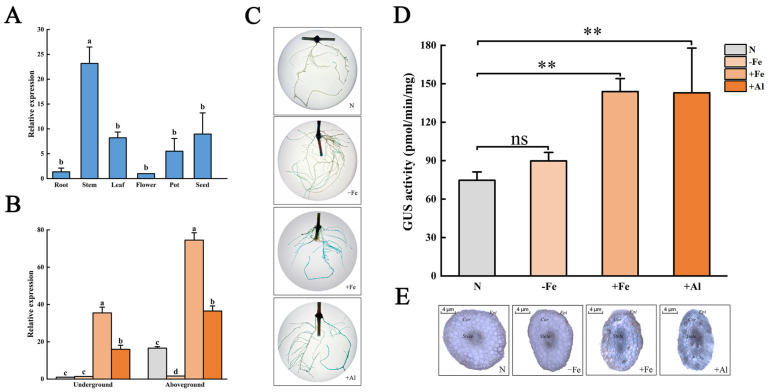
Response of *GmCSY3* to Fe deficiency, excess Fe, and Al stresses. (**A**) The expression level of *GmCSY3* in the root, stem, leaf, flower, pod, and seed of soybean Heinong 51. (**B**) The expression of the *GmCSY3* gene in the aboveground and underground parts of soybean under Fe deficiency, excess Fe, Al, and control. (**C**) GUS staining of pBI121-*GmCSY3*pro::GUS transformed soybean hairy roots. (**D**) GUS enzyme activity of pBI121-*GmCSY3*pro::GUS transformed soybean hairy roots. (**E**) A cross-section of soybean hairy roots. Statistical significance is denoted by ** *p* < 0.01. Lowercase letters indicate *p* < 0.05. ns represents no significant difference.

To detect the tissue characteristics and the responses to stress of *GmCSY3*, we conducted cis-element analysis of qRT-PCR and GUS assay using soybean hairy roots. The 1616 bp *GmCSY3* promoter (*GmCSY3*pro) was cloned from HN51 (Supplementary Figure S2), and the cis element of *GmCSY3*pro is shown in Supplementary Table S2. Bioinformatics predicted that many response elements existed in the promoter of *CSY* genes. Among them, the promoters of *GmCSY3* and *GmCSY9* are clustered into one category (Figure 2A). Multiple elements related to growth and development, and many elements related to biological and abiotic stress were found in the *GmCSY3*pro (Figure 2B). The qRT-PCR showed that *GmCSY3* was expressed in both vegetative organs (root, stem, and leaf) and reproductive organs (flower, pod, and seed) of soybean. The expression levels from highest to lowest were as follows: stem, seed, leaf, pod, root, and flower (Figure 3A). Compared to the control (Normal treatment, N), the expression of *GmCSY3* showed no significant difference in the underground part, while it was significantly decreased in the aboveground part of soybean under Fe deficiency (−Fe) (*p* < 0.05); the expression of *GmCSY3* significantly increased in underground and aboveground parts of soybean under excessive Fe stress (+Fe) and Al stress (+Al) (*p* < 0.05); the expression of *GmCSY3* was significantly higher under +Fe than that under +Al, respectively, in underground and aboveground parts of soybean (*p* < 0.05) (Figure 3B).

The pBI121-*GmCSY3*pro::GUS recombinant expression vector was constructed and transformed into soybean (Supplementary Figures S3 and S4). The soybean hairy roots were treated with −Fe, +Fe, or +Al and N. The hairy roots presented deeper staining under +Fe and +Al while showing no significant difference under −Fe compared with the control (Figure 3C). The GUS activity was significantly higher in soybean hairy roots under +Fe and +Al, while showing no significant difference under −Fe, compared with the control (*p* < 0.01) (Figure 3D). The hairy roots’ transection showed that cortical tissue was lightly stained, and the vascular tissue, including phloem and xylem, was deeply stained in both treatments and the control. Under +Fe and +Al treatment, the staining was deep, with the deep staining area scattered in the cortex. No significant difference was found between the −Fe treatment and the control (Figure 3E). The results indicated that the *GmCSY3* gene expression was induced by +Fe and +Al stress, most strongly by +Fe, but was not induced by −Fe stress.

### 3.3. The +Fe and +Al Stress Resistance of GmCSY3 Heterologous Expressed Yeast

**Figure 4:**
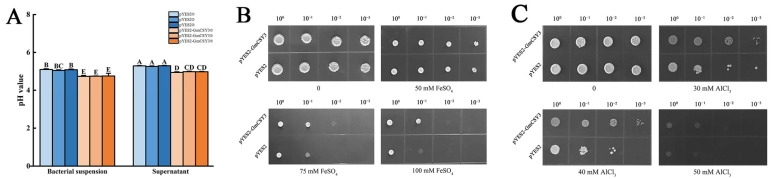
The growth of *GmCSY3* recombinant yeast under excess Fe and Al stresses. (**A**) The pH value of the *GmCSY3* recombinant yeast of bacterial suspension and supernatant. (**B**) The growth of *GmCSY3* recombinant yeast under different concentrations (0 mM, 10 mM, 50 mM, and 100 mM) of FeSO_4_ treatment. (**C**) The growth of *GmCSY3* recombinant yeast under different concentrations (0 mM, 30 mM, 40 mM, and 50 mM) of AlCl_3_ treatment, assessed by spotting serial tenfold dilutions (from 10^0^ to 10^−4^) of yeast cultures on media. Capital letters indicate *p* < 0.01.

The recombinant vector pYES2-*GmCSY3* was constructed and transformed into *Saccharomyces cerevisiae* INVSc1. The pH values of the bacterial liquid before and after centrifugation were compared. In both cases, the pH value of the control (pYES2 transformed yeast) was significantly higher than that of the *GmCSY3* recombinant yeast (Figure 4A). *GmCSY3* recombinant yeast and the control (pYES2 transformed yeast) were treated with +Fe and +Al stress, respectively. Under different gradient concentration treatments, both transgenic yeast and the control yeast showed a trend of growth destruction as the treatment concentration increased. Under normal conditions, the growth of the *GmCSY3* recombinant yeast and the control showed no significant difference. There was no difference in growth between the *GmCSY3* recombinant yeast and the control under 50 mM FeSO_4_ treatment at different concentrations of dilution. *GmCSY3* recombinant yeast grew, while the control did not grow when treated with 75 mM FeSO_4_ and diluted to 10^−2^. *GmCSY3* recombinant yeast grew, while the control did not grow with 100 mM FeSO_4_ diluted to 10^−1^ (Figure 4B). *GmCSY3* recombinant yeast grew, while the control yeast did not grow when treated with 30 mM AlCl_3_ diluted to 10^−3^, 40 mM AlCl_3_ diluted to 10^−3^, and 0 mM AlCl_3_ diluted to 10^−1^ (Figure 4C). The results showed that *GmCSY3* increased the resistance of yeast to Fe and Al stress.

### 3.4. The +Fe and +Al Stress Resistance of GmCSY3 Overexpressed and RNAi-Suppressed Soybean Transformation Chimeras

To determine the function of *GmCSY3*, the overexpression and RNAi vectors were constructed. The *GmCSY3* was driven by the 35S promoter in the overexpression vector pBI121-*GmCSY3*. The expression of *GmCSY3* was inhibited by introducing the 541 bp sequence of *GmCSY3* by the RNAi vector pZH01-*GmCSY3* (Supplementary Figures S5 and S6). The vectors were transformed into *Agrobacterium tumefaciens* K599 and then transformed into soybean, respectively (Supplementary Figures S7 and S8). The soybean young seedlings were transformed by cotyledon injection, and three kinds of transgenic chimera lines were obtained, named as OE (*GmCSY3* overexpressed lines), RNAi (*GmCSY3* inhibited lines), and Ev (K599 transformed without vector) (Supplementary Figure S9).

**Figure 5:**
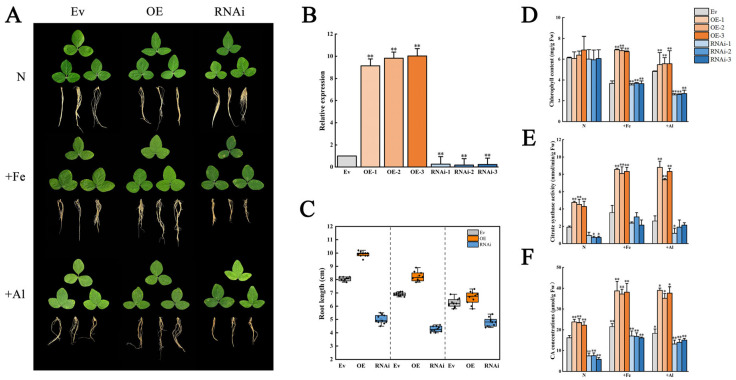
The phenotype of transgenic soybean chimeras under excess Fe and Al stresses. The OE, RNAi, and Ev soybean chimeras were obtained by transformation of the *GmCSY3* overexpression vector, *GmCSY3* RNAi-suppressed vector, and the empty vector, respectively. (**A**) Phenotype. (**B**) *GmCSY3* expression in the hairy roots. (**C**) Root length. (**D**) Chlorophyll content. (**E**) Citrate synthase activity. (**F**) CA content. Statistical significance is denoted by * *p* < 0.05 and ** *p* < 0.01.

To detect the effect of *GmCSY3* on the resistance to +Fe and +Al stresses, the transformed lines were treated with +Fe and +Al. Under normal conditions, the leaf phenotype and chlorophyll content showed no significant difference among OE, RNAi, and Ev. Under +Fe or Al stress, the leaves were yellow, and chlorophyll contents were decreased in all three chimeras plants, compared to normal conditions. The leaves were greener in OE and more yellow in RNAi, compared to those of Ev (Figure 5A). The expression of *GmCSY3* in OE hairy roots was significantly higher than that of Ev (*p* < 0.01) and was significantly lower than that of Ev (*p* < 0.01) (Figure 5B). Compared to Ev, the roots were significantly longer in OE and shorter in RNAi under normal conditions (*p* < 0.01). Under +Fe or +Al stresses, all of the root lengths were decreased. Compared to Ev, the roots were significantly longer in OE and shorter in RNAi under +Fe stress; the roots were significantly shorter in RNAi (*p* < 0.01) while showing no difference in OE under +Al stress (Figure 5C). The chlorophyll content was significantly higher in OE, while it was significantly lower in RNAi compared to that of Ev, respectively (*p* < 0.01) (Figure 5D). Under normal conditions, the citrate synthase activity was significantly higher than that of Ev (*p* < 0.01) and was significantly lower than that of Ev in RNAi-2 and RNAi-3 (*p* < 0.05). Under +Fe and +Al stresses, the citrate synthase activity was significantly higher than that of Ev (*p* < 0.01) and was significantly lower than that of Ev, only significantly in RNAi-1 under +Al stress (*p* < 0.05) (Figure 5E). Under the three treatment conditions (N, +Fe, +Al), the CA concentration in the OE group was significantly higher than that in the Ev group (*p* < 0.01), while the CA concentration in the RNAi group was significantly lower than that in the Ev group (*p* < 0.01) (Figure 5F). The results showed that expression of *GmCSY3* directly affected CAS activity and CA concentration. Together, the changes in citrate synthase activity resulting from *GmCSY3* manipulation correlated with the altered tolerance to Fe/Al stress (e.g., chlorophyll content and root growth), establishing *GmCSY3*’s role in stress response through citrate synthesis.

### 3.5. The ROS and Protective Enzymes Activity of GmCSY3 Overexpressed and RNAi-Suppressed Soybean Transformation Chimeras Under +Fe or Al Stress

**Figure 6:**
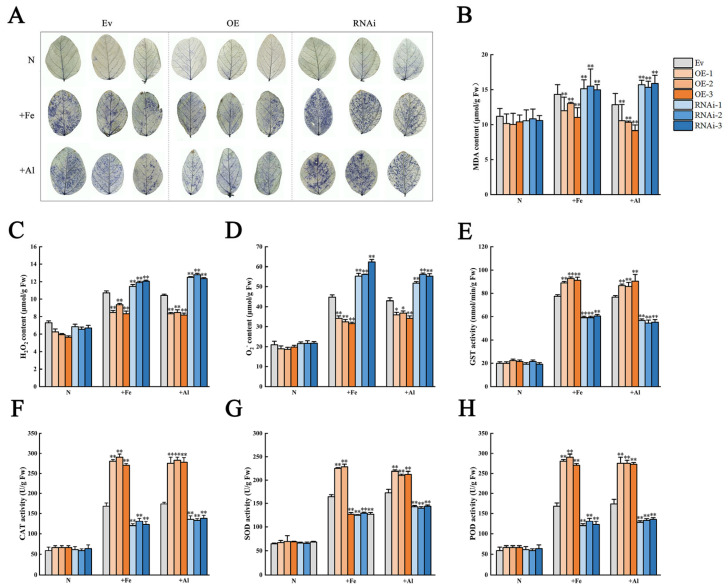
The physiological indicators of transgenic soybean chimeras under excess Fe and Al stresses. The OE, RNAi, and Ev soybean chimeras were obtained by transformation of the *GmCSY3* overexpression vector, the *GmCSY3* RNAi-suppressed vector, and the empty vector, respectively. (**A**) NBT staining. (**B**) MDA content. (**C**) H_2_O_2_ content. (**D**) O_2_^−^ content. (**E**) GST activity. (**F**) CAT activity. (**G**) SOD activity. (**H**) POD activity. Statistical significance is denoted by * *p* < 0.05 and ** *p* < 0.01.

To determine the effect and mechanism of *GmCSY3* against metal toxicity, we examined the degree of oxidative damage and the activity of protective enzymes in transgenic lines under +Fe or +Al stresses. Under normal conditions, the NBT staining showed no significant difference in the leaves of OE, RNAi, and Ev. Under +Fe or +Al stresses, the NBT staining was distinctly lighter in the leaves of OE and significantly darker in the leaves of RNAi than that of Ev (Figure 6A). Under normal conditions, MDA, H_2_O_2_, and O_2_^−^ contents showed no significant difference in the roots of OE, RNAi, and Ev. Under +Fe stress, MDA, H_2_O_2_, and O_2_^−^ contents were significantly lower in the roots of OE and significantly higher in the roots of RNAi than those of Ev (*p* < 0.01). Under +Al stress, MDA content was significantly lower in the roots of OE and RNAi than that of Ev (*p* < 0.01). H_2_O_2_ and O_2_^−^ contents were significantly lower in the roots of OE (*p* < 0.01, 0.05) and significantly higher in the roots of RNAi than those of Ev (*p* < 0.01) (Figure 6B–D). Under normal conditions, the activity of GST, CAT, SOD, and POD showed no significant difference in the roots of OE, RNAi, and Ev. Under +Fe or +Al stresses, the activity of GST, CAT, SOD, and POD was significantly lower in the roots of OE and significantly higher in the roots of RNAi than that of Ev (*p* < 0.01) (Figure 6E–H). The results showed that the overexpression of the *GmCSY3* gene reduced the oxidative damage caused by +Fe and +Al stress, and improved the plant tolerance to excess Fe and Al stress.

### 3.6. The Distribution of Fe and Al in the Roots of GmCSY3 Overexpressed and Inhibition-Expressed Soybean Transformation Chimeras

**Figure 7:**
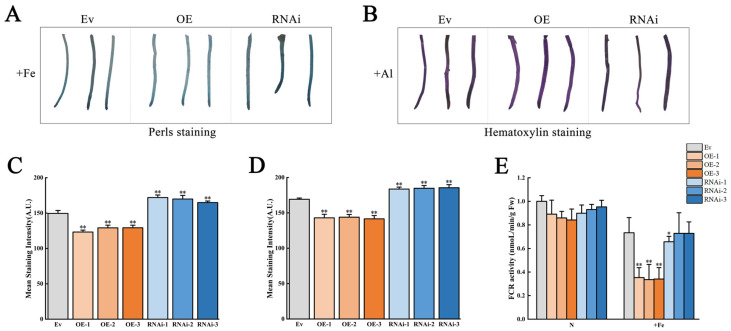
The FCR activity and distribution of Fe and Al in the roots of transgenic soybean chimeras under excess Fe and Al stresses. The OE, RNAi, and Ev soybean chimeras were obtained by transformation of the *GmCSY3* overexpression vector, the *GmCSY3* RNAi-suppressed vector, and the empty vector, respectively. (**A**) Perls staining. (**B**) Hematoxylin staining. (**C**) Perls’ mean staining intensity. (**D**) Hematoxylin mean staining intensity. (**E**) FCR activity. Statistical significance is denoted by ** *p* < 0.01and * *p* < 0.05.

Under +Fe stress, Perls staining showed that the roots of OE were lighter and the roots of RNAi were deeper than those of Ev (Figure 7A); Under +Al stress, Hematoxylin staining showed that the roots of OE were lighter purple and the roots of RNAi were deeper than those of Ev (Figure 7B); Perls staining mean intensity analysis showed that the staining intensity was the lowest in the OE group, followed by the K599 group, while the RNAi group had the highest intensity (*p* < 0.01) (Figure 7C). Quantitative assessment of hematoxylin staining mean intensity revealed a distinct trend: staining intensity was the lowest in the OE group, moderate in the K599 group, and peaked in the RNAi group. (*p* < 0.01) (Figure 7D). To better understand the influence of *GmCSY3* on the absorption of Fe and Al, the activity of FCR enzyme and the distribution of Fe and Al in transgenic plants were examined. Under normal conditions, FCR activity showed no significant difference in the roots of OE, RNAi, and Ev. Under +Fe stress, the FCR activity significantly decreased in the roots of OE, compared to that of Ev (*p* < 0.01); the FCR activity only significantly decreased in the roots of RNAi-1, but not in the roots of RNAi-2 and RNAi-3, compared to that of Ev (*p* < 0.05) (Figure 7E).

## 4. Discussion

The molecular characterization and expression analysis presented herein provide significant insights into the potential role of *GmCSY3* in physiology and metal stress response, particularly concerning Fe and Al homeostasis. Our bioinformatics analysis firmly established *GmCSY3* as a member of the *CSY* gene family in soybean. The evolutionary relationship and high similarity suggest conservatism in function among leguminous *CSY* genes [33,81]. The tandem duplicate of *GmCSY3* with *GmCSY9* and *GmCSY6* strongly suggests functional redundancy or subfunctionalization among these *GmCSYs*. We observed that *GmCSY3* is more closely related to *GmCSY9* but has greater differences with *GmCSY6* in terms of gene structure and cis-elements in the promoter. This reflects the evolution driven by gene replication, which is very likely to lead to the divergence of *GmCSY* genes involved in diverse metabolic pathways or stress responses. Bioinformatics has predicted the mitochondrial region (see Figure 1E), which is a typical feature of the CSY protein. However, the current experiments and the provided localization information of the GmCSY3 protein in the cytoplasm have been completed. Citric acid plays an important role in the tricarboxylic acid cycle and may be a potential exit for chelation. Confirming GmCSY3’s localization will help deepen our understanding of this mechanism, and future research directions need to be supplemented. In the future, we will verify the subcellular localization of this protein by performing fluorescence labeling co-localization experiments and combining with homologous or heterologous expression systems.

The citrate synthases were thought to be a housekeeping enzyme early, but with further study, gene expression of citrate synthase genes showed tissue dependence and a specific response to stress. The numerous elements in *GmCSY3*pro indicated that *GmCSY3* expression was associated with both developmental processes and abiotic/biotic stress responses. The expression of *GmCSY3* across major vegetative and reproductive organs suggests broad roles in primary metabolism beyond the TCA cycle, potentially involving organic acid provision for biosynthetic pathways, pH regulation, or nutrient loading. The high expression of *GmCSY3* in stems observed in this study, similar to *CSY* genes in Arabidopsis [82] and *RmCSs* in *Secale cereale* [36], suggested its role in phloem loading or long-distance transport of citrate-specifying metals, based on the known functions of *CS*.

The low expression in the roots under normal conditions is different from that of *CSY* genes in roots of *petunia*, Arabidopsis, rye, short-stemmed chrysanthemum and several fruit trees, which are usually associated with the metal micronutrients absorption to address the nutrients deficiency or secrete CS into the rhizosphere to chelate and fix toxic metal elements, thereby helping plant growth and minimizing the toxicity to plants [45,46]. *CS* genes in trees are induced by iron deficiency and decrease after sufficient citrate accumulation or excessively high iron concentration. Our research indicated that *GmCSY3* was lowly expressed in roots, corresponding to its non-response to −Fe, suggesting that the *GmCSY3* gene is different from the above-mentioned *CSY* genes, and has rather special functions. Further, the result is corroborated by the abundance of *GmCS* mRNA, which did not show significant changes related to iron deficiency [83], which reflects the divergent differentiation of homologous *CSY* genes among different species. The lack of response to −Fe also acknowledges that citric acid is not the main response mode for iron acquisition. Strategy 1 of non-grass plants, at least *GmCSY3*, has not made a significant contribution in this regard. Significant upregulation of *GmCSY3* in roots and shoots by +Fe and +Al stresses demonstrated that *GmCSY3* might play a primary role for *GmCSY3* in mitigating metal toxicity by storage or transportation, rather than a general response to nutrient deficiency. The intense GUS staining, particularly localized within the vascular tissues (xylem and phloem) and scattered within the cortex under +Fe and +Al (Figure 3E,F), reinforces this interpretation. Al stress significantly upregulates *GmCSY3* in soybean roots and leaves to support the induction of citrate synthase, which is a tolerance mechanism for aluminum in many plants, including some leguminous plants [49,84,85,86]. This result supported the idea that the *CS* gene expression induced by Al stress also varied according to species or genes.

The *GmCSY3* heterologous expressed yeast provides compelling evidence in the improvement in tolerance to excess iron and aluminum stress, with enhanced acid production and medium acidification. As citrate synthase catalyzes the committed step in citrate biosynthesis, this measurable acidification strongly suggests that GmCSY3 is enzymatically active within the yeast system and drives the production and subsequent secretion of citrate anions into the extracellular environment. This finding provides a direct mechanistic link between *GmCSY3* expression and a key process—organic acid efflux—known to be central to metal chelation and detoxification in plants and other organisms. These results also provide new ideas for the cross-species use of the *GmCSY3* gene.

The generation of composite soybean plants provides critical in planta functional validation for the role of *GmCSY3* in conferring tolerance to excess Fe and Al toxicity. This experimental system directly links *GmCSY3* expression levels to citrate synthase (CS) activity, physiological stress responses, and underlying mechanisms of oxidative stress mitigation. The robust correlation between *GmCSY3* transcript abundance and citrate synthase activity across genotypes under both control and stress conditions provides definitive evidence that *GmCSY3* encodes a catalytically active CS isoform (Figure 5B,C).

Fe accumulation is extremely toxic to plants and affects the entire metabolic process of plants [87]. Excess Fe toxicity increases the production of hydroxyl free radicals through the Fenton reaction, resulting in strong oxidative stress, macromolecular damage, enzyme inactivation, pigment damage, reduced photosynthetic activity, low carbon fixation rate, and reduced biomass accumulation [88,89,90]. Unlike Fe, Al is not necessary for most living things. In plant cells, the root is damaged first under Al stress, and the root is the main damaged site [91,92]. GmCSY3 activity significantly influenced root growth under stress, demonstrating that basal levels of GmCSY3-derived citrate are essential for root growth maintenance under Al stress and a direct benefit of citrate in mitigating excess Fe toxicity within root tissues and/or the rhizosphere. Leaf damage may also be caused by the indirect influence of Fe accumulation in roots [93]. *GmCSY3* provides significant protection against leaf chlorosis, indicating that enhanced citrate synthesis in roots directly mitigates metal toxicity impacts on shoot physiology.

Excess Fe and Al induce toxicity largely through the generation of reactive oxygen species (ROS) [94]. Histochemical staining demonstrates that GmCSY3 activity effectively reduces H_2_O_2_ production and oxidative damage induced by Fe and Al toxicity. The contents of H_2_O_2_ and O_2_^−^ under +Fe stress and H_2_O_2_ and O_2_^−^ under Al stress also indicate that the generation and accumulation of ROS significantly decrease in OE but increase in RNAi. Correspondingly, MDA content is significantly lower in OE, and significantly higher in RNAi, indicating that membrane lipid peroxidation is reduced by *GmCSY3* overexpression and exacerbated by *GmCSY3* expression suppression. The activity of key antioxidant enzymes (GST, CAT, SOD, POD) in roots showed a consistent pattern, suggesting that plants with OE roots experience less severe oxidative stress, consequently requiring less induction of their enzymatic antioxidant defense systems. The elevated activity in RNAi roots reflects a more acute oxidative stress response, indicating that these plants are struggling to cope with the heightened ROS burden resulting from reduced citrate-mediated protection [95].

FCR is usually induced under iron deficiency conditions to promote the reduction of Fe^3+^ and subsequent uptake [96]. FCR activity was significantly lower in OE roots when treated with excess Fe, indicating that plants reduce the absorption of iron from the rhizosphere by decreasing FCR activity. In the same excess Fe culture, the staining results indicated that the free Fe^3+^ in OE roots was less than that in RNAi and the control. Although the FCR enzyme activity of RNAi under excess Fe was not significantly higher than that of the control, more Fe^3+^ was clearly observed in the staining of RNAi. The reasons for the difference in the accumulated Fe^3+^ may be as follows: First, CS enzymes catalyze the generation of citric acid, and the citrate derived from it chelates Fe^3+^, resulting in less free Fe^3+^ that can combine with the staining solution and chromogenic reaction. Second, citrate chelates Fe^3+^ to transport iron from the underground part to the above-ground part, thereby reducing the iron content in the roots. Third, Citric acid synthesis is involved in plants’ perception of the iron status in their surrounding environment, and regulates the key enzyme FCR for iron absorption. Therefore, when *GmCSY3* is upregulated, plants are more sensitive to iron overload. On the one hand, chelate and sequester Fe^3+^ or transport them to tissues that require iron. On the other hand, produce iron rejection behavior such as negatively regulating FCR, to prevent more iron uptake and cause toxicity. When *GmCSY3* is downregulated, the plants’ ability to chelate and transport Fe^3+^ was insufficient, and their perception of iron overload and iron absorption was not affected, remaining the same as those of the control plants. Under +Al stress, the free Al^3+^ in OE roots was lower, while it was higher in RNAi roots. This finding is consistent with the role of citrate in Al detoxification. The citrate in the cytoplasm chelates and internalizes Al^3+^ to form less toxic chimeras. Knockdown of *GmCSY3* affects the production of citrate, leading to an increase in Al accumulation, which will overwhelm the cell’s detoxification ability and cause severe toxicity [94].

Based on our research results of *GmCSY3* can regulate citrate accumulation and is involved in iron homeostasis. We will focus on the following research directions in future studies: to determine whether citric acid transporters such as MATE and ALMT are related to the function of *GmCSY3* and explore the influence of hormones on *GmCSY3* expression. *GmCSY3* is likely involved in the iron homeostasis regulation mechanism mediated by *FIT*, *IRT1*, or ethylene/NO-related factors, thereby regulating iron absorption and homeostasis maintenance in soybeans. We will further study to explore the *GmCSY3* revealed regulatory mechanisms. Through detailed research in these aspects, we deepen the understanding of the *GmCSY3* gene and provide more detailed information for its application.

## 5. Conclusions

This study provides conclusive genetic evidence that *GmCSY3* encodes a functional citrate synthase, which is crucial for soybeans’ tolerance to excessive Fe and Al. The gene expression of *GmCSY3* responds to excessive Fe and Al and can enhance the yeast’s tolerance to excessive Fe and Al. Particularly important, *GmCSY3* alleviates oxidative stress and physiological damage induced by excessive Fe and Al through promoting citrate synthesis and chelating toxic metal ions. We also demonstrated that *GmCSY3* is involved in the modulation of iron homeostasis, generating signal molecules that help plants perceive the stress state within cells and modulate the iron absorption pathways of plants. The role of *GmCSY3* in modulating Fe and Al accumulation and protective effect highlights its core role in soybean adaptation and its great potential as a target for crop improvement.

## Data Availability

The original contributions presented in this study are included in the article. Further inquiries can be directed to the corresponding authors.

## Supplementary Materials

The following supporting information can be downloaded at: https://www.mdpi.com/article/10.3390/biology15010105/s1, Supplementary Table S1: Information of primers used in this study; Supplementary Table S2: The cis-elements in *GmCSY3* Promoter; Figure S1: PCR amplification of *GmCSY3* gene; Figure S2: PCR amplification of *GmCSY3*pro; Figure S3: Identification of recombinant plasmids pBI121-*GmCSY3*pro::GUS by *Hind III* and *Xba I*; Figure S4: The PCR amplification product of transformed Agrobacterium (K599) with pBI121-*GmCSY3*pro::GUS; Figure S5: Schematic diagram of the construction of overexpression vector pBI121-*GmCSY3*; Figure S6: Schematic diagram of the construction of RNAi vector pZH01-*GmCSY3*; Figure S7: Bacterial colony PCR identification of construction of plant expression vector pBI121-*GmCSY3*; Figure S8: Bacterial colony PCR identification of construction of RNAi vector pZH01-*GmCSY3*; Figure S9: Transgenic soybean hairy roots with *GmCSY3*.
