# Supplementary material for: Overexpression of GmCSY3 Enhances Soybean Tolerance to Excess Iron and Aluminum
Source: Biology (Basel). 2026 Jan 5;15(1):105. doi: 10.3390/biology15010105 (PMC12785149; doi:10.3390/biology15010105)
Supplement: Supplementary file 1 [file biology-15-00105-s001.zip › Supplementary Materials.pdf]

Supplementary Table S1. Information of primers used in this study.

Primer	Sequence (5′-3′)	Purpose
GmCSY3 S	5′- GCTCTAGAATGGCATTCTTCAGAAGCGTT -3′	Cloning of *GmCSY3*
GmCSY3 A	5′- TCCCCCGGGTCAGGATGATTTGGCGACC -3′	
GmACTIN S	5′- ACGAGCGTTTCAGATG-3′	Quantitative RT PCR
GmACTIN A	5′- ACCTCCGATCCAGACA-3′	
*GmCSY3*q S	5′- CAGGGACAATACAACAGAAGA-3′	
*GmCSY3*q A	5′- GTGAAATCCATTCCAGCATAC-3′	
*GmCSY3*pro S	5′- CTGAAAGGCAAACGAATC-3′	Cloning of *GmCSY3* promoter
*GmCSY3*pro A	5′- AAGAATGCCATTTGCG-3′	
GmCSY3-1 S	5′- CGGGATCCTTTCTGCTCATCCAATGACTC -3′	RNAi
GmCSY3-1 A	5′- GCTCTAGATGTGTGTGAACTAACATTGCC -3′	
GmCSY3-2 S	5′- CGAGCTCTTTCTGCTCATCCAATGACTC -3′	
GmCSY3-2 A	5′- GGGTACCTGTGTGTGAACTAACATTGCC -3′	

Supplementary Table S2. The cis-elements in *GmCSY3* Promoter.

**Classification**	**Name**	**Function Describe**	**Number**
The core components	CAAT-box	common cis-acting element in promoter and enhancer regions	23
	TATA-box	core promoter element around -30 of transcription start	25
	ARE	cis-acting regulatory element essential for the anaerobic induction	2
	AT-TATA-box	data	1
Hormone responsive element	ABRE	cis-acting element involved in the abscisic acid responsiveness	2
	CGTCA-motif	cis-acting regulatory element involved in the MeJA-responsiveness	1
	TGACG-motif	cis-acting regulatory element involved in the MeJA-responsiveness	1
	as-1	MeJA	1
	TATC-box	cis-acting element involved in gibberellin-responsiveness	1
	TCA-element	cis-acting element involved in gibberellin-responsiveness	2
Light responsive element	AF1 binding	light responsive element	1
	ACE	cis-acting element	1
	AE-box	involved in light responsiveness	1
	Box4	part of a conserved	1
	CAG-motif	DNA	1
	Box	part of a light response element	1
		cis-acting regulatory element	

	G-box	involved in light responsiveness	2
		cis-acting regulatory element involved in light responsiveness	
	TCT-motif	part of a light responsive element	2
	Gap-box	part of a light responsive element	1
	GT1-motif	light responsive element	1
	MYB	protein binding site	2
	Box III		1
	E-Box		2
	ERE		1
	LTR	cis-acting element involved in low-temperature responsiveness	1
Other element	MSA-like	cis-acting element involved in cell cycle regulation	1
	MYC		2
	WUN-motif		1
	WRE3		1
	W box		3
	AAGAA-motif		5
Unnamed element	Unnamed_1		2
	Unnamed_4		5

Supplementary Figure S1. PCR amplification of *GmCSY3* gene

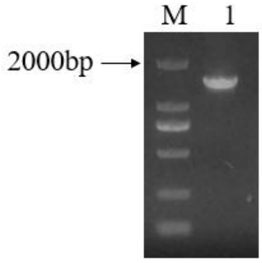

M, DL 2000; 1, *GmCSY3* gene

Supplementary Figure S2. PCR amplification of *GmCSY3*pro

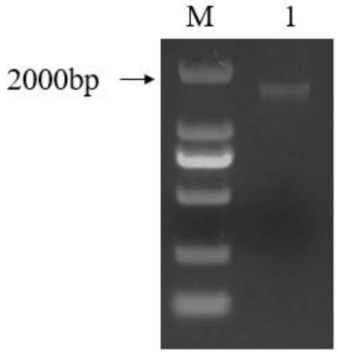

M, DL 2000; 1, *GmCSY3*pro

Supplementary Figure S3. Identification of recombinant plasmids pBI121-*GmCSY3*pro::GUS by *Hind III* and *Xba I*

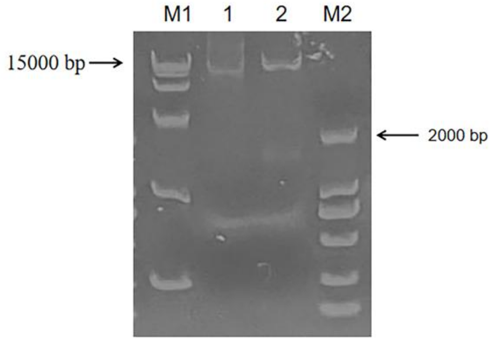

M1, Maker DL15000; M2, Maker DL 2000; 1, Recombinant plasmids pBI121-*GmCSY3*pro::GUS; 2, *Hind III* and *Xba I* digestion products

Supplementary Figure S4. The PCR amplification product of transformed Agrobacterium (K599) with pBI121-*GmCSY3*pro::GUS

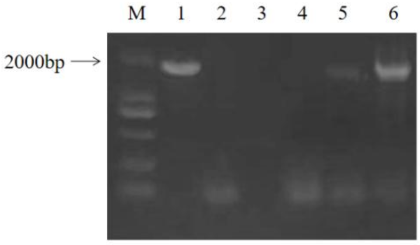

M, DL 2000; 1, pBI121-*GmCSY3*pro::GUS plasmid; 2, Negative control; 3, blank control; 4, colony1; 5, colony2; 6, colony3

Supplementary Figure S5. Schematic diagram of the construction of overexpression vector pBI121-*GmCSY3*

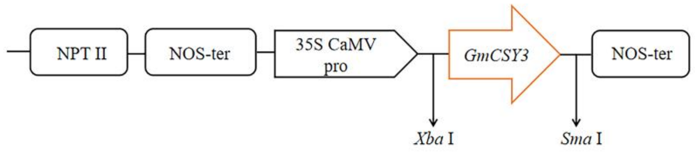

Supplementary Figure S6. Schematic diagram of the construction of RNAi vector pZH01-*GmCSY3*

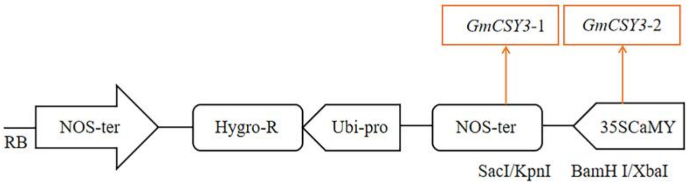

Supplementary Figure S7. Bacterial colony PCR identification of construction of plant expression vector pBI121-*GmCSY3*

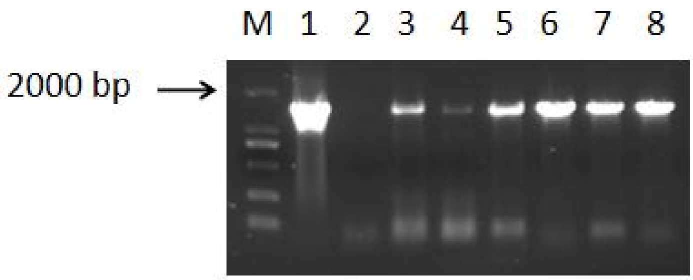

M, DL 2000; 1, Positive control; 2, Negative control; 3, Blank control; 4, colony1; 5, colony2; 6, colony3

Supplementary Figure S8. Bacterial colony PCR identification of construction of RNAi vector pZH01-*GmCSY3*

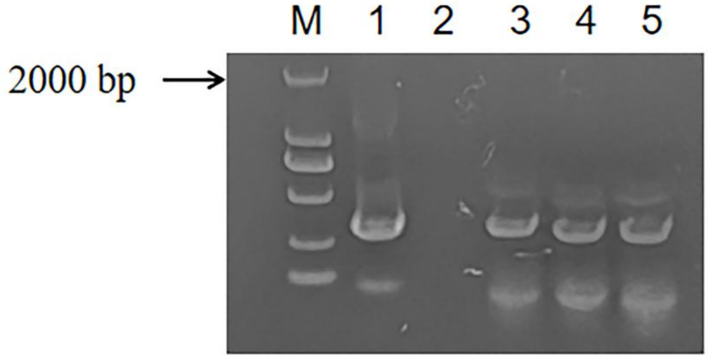

M, DL 2000; 1, Positive control; 2, Negative control; 3, Blank control; 4, colony1; 5, colony2; 6, colony3; 7, colony4

Supplementary Figure S9. Transgenic soybean hairy roots with *GmCSY3*

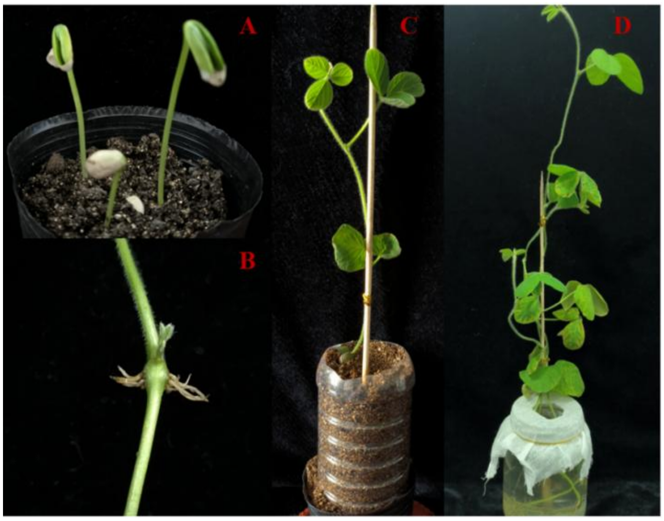

A, Soybean seedlings with unfolded cotyledon; B, Hairy roots appeared at the infection site; C, Vermiculite culture soybean hairy roots; D, Grow hairy roots：E, Hairy root of hydroponic soybean
