# Supplementary figures and images for: Overexpression of GmCSY3 Enhances Soybean Tolerance to Excess Iron and Aluminum
Source: Biology (Basel). 2026 Jan 5;15(1):105. doi: 10.3390/biology15010105 (PMC12785149; doi:10.3390/biology15010105)

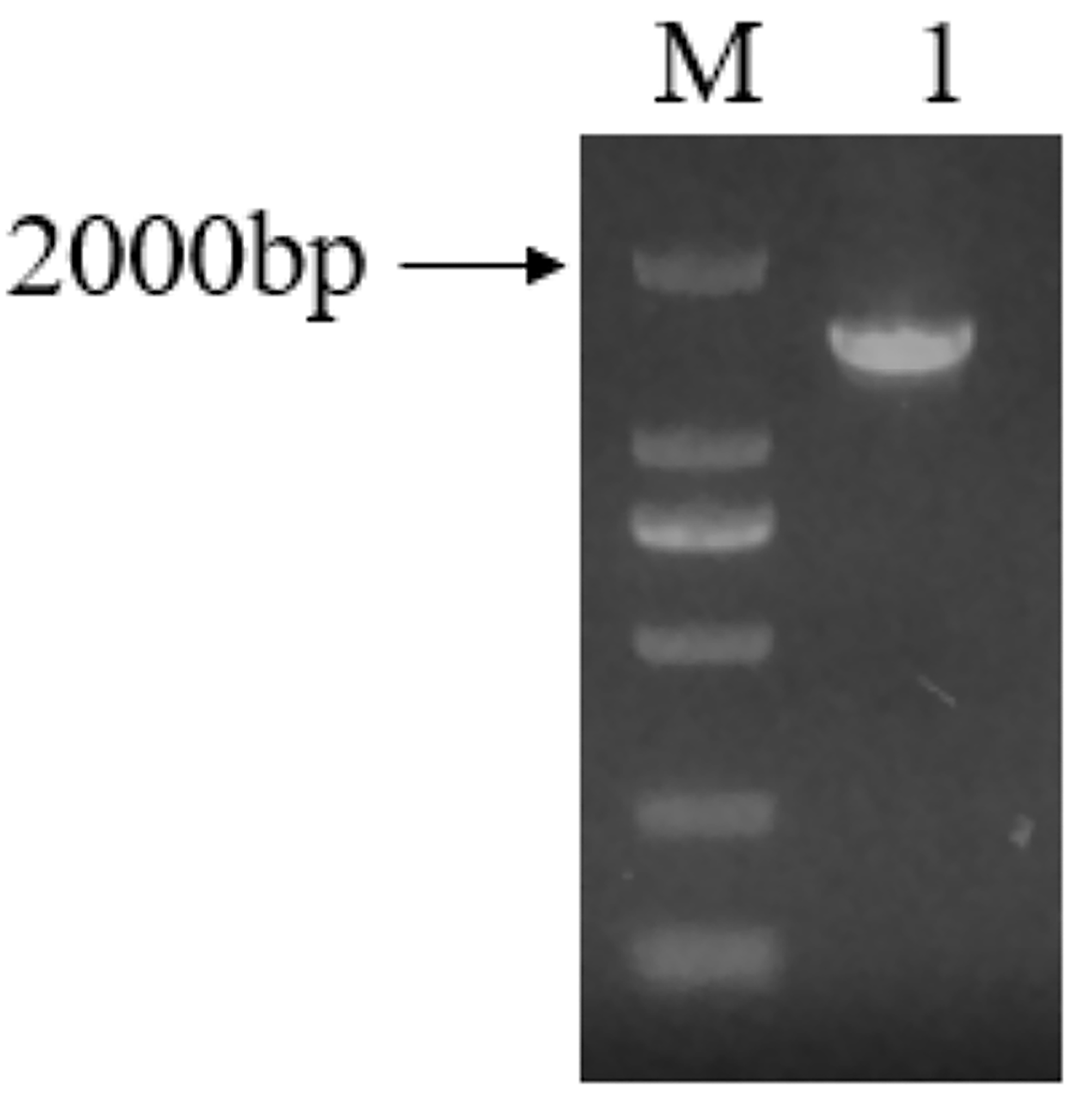

Supplement: Supplementary file 1 [file biology-15-00105-s001.zip › Supplementary Figure S1. PCR amplification of GmCSY3 gene.tif]

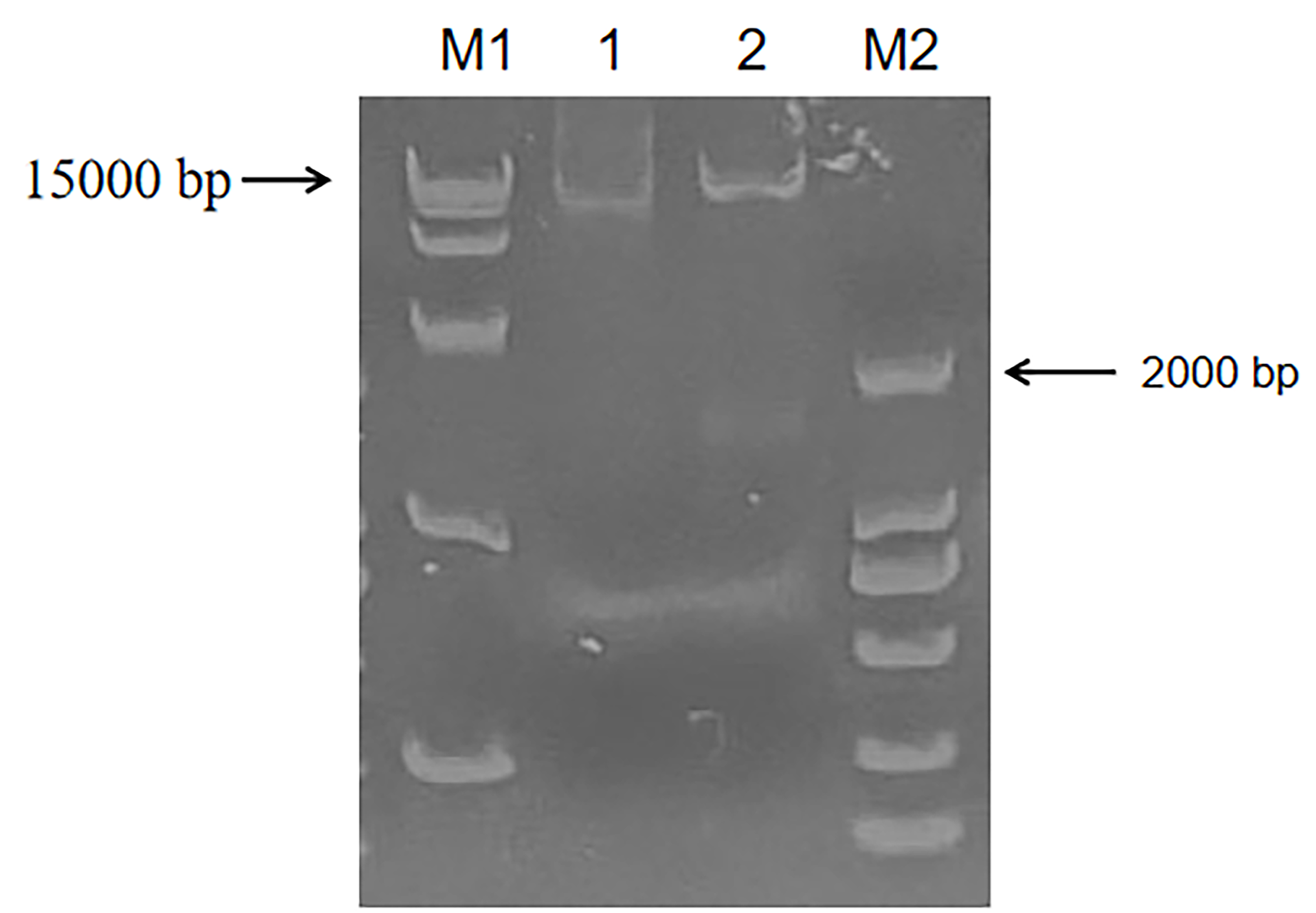

Supplement: Supplementary file 1 [file biology-15-00105-s001.zip › Supplementary Figure S3. Identification of recombinant plasmids pBI121-GmCSY3proGUS by Hind III and Xba I.tif]

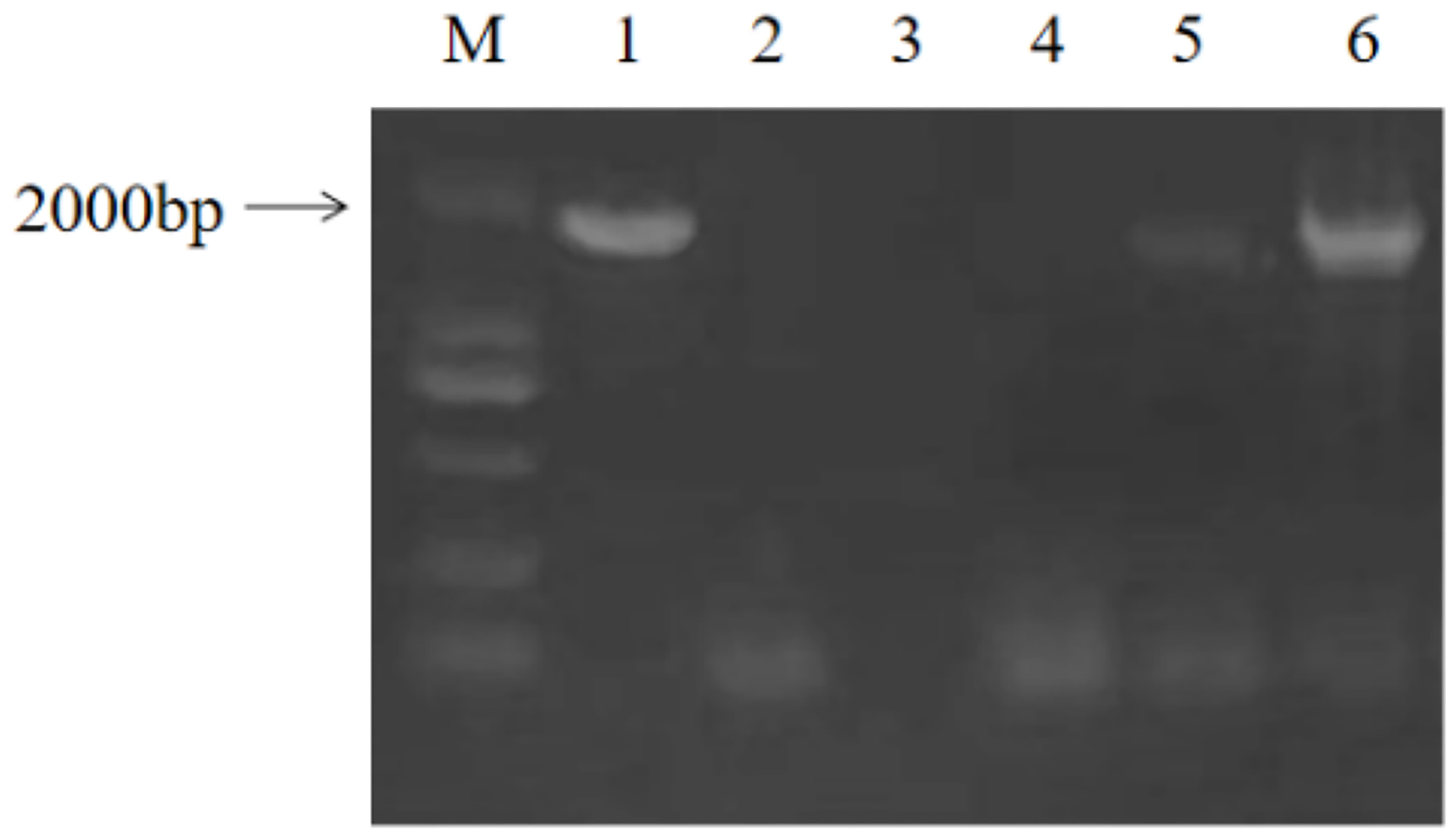

Supplement: Supplementary file 1 [file biology-15-00105-s001.zip › Supplementary Figure S4. The PCR amplification product of transformed Agrobacterium (K599) with pBI121-GmCSY3proGUS.tif]

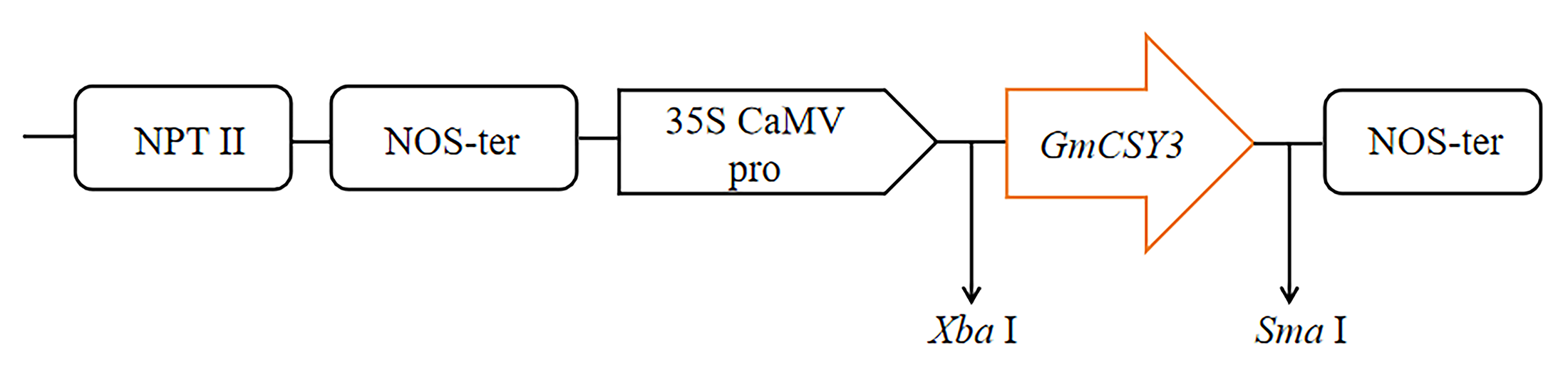

Supplement: Supplementary file 1 [file biology-15-00105-s001.zip › Supplementary Figure S5. Schematic diagram of the construction of overexpression vector pBI121-GmCSY3.tif]

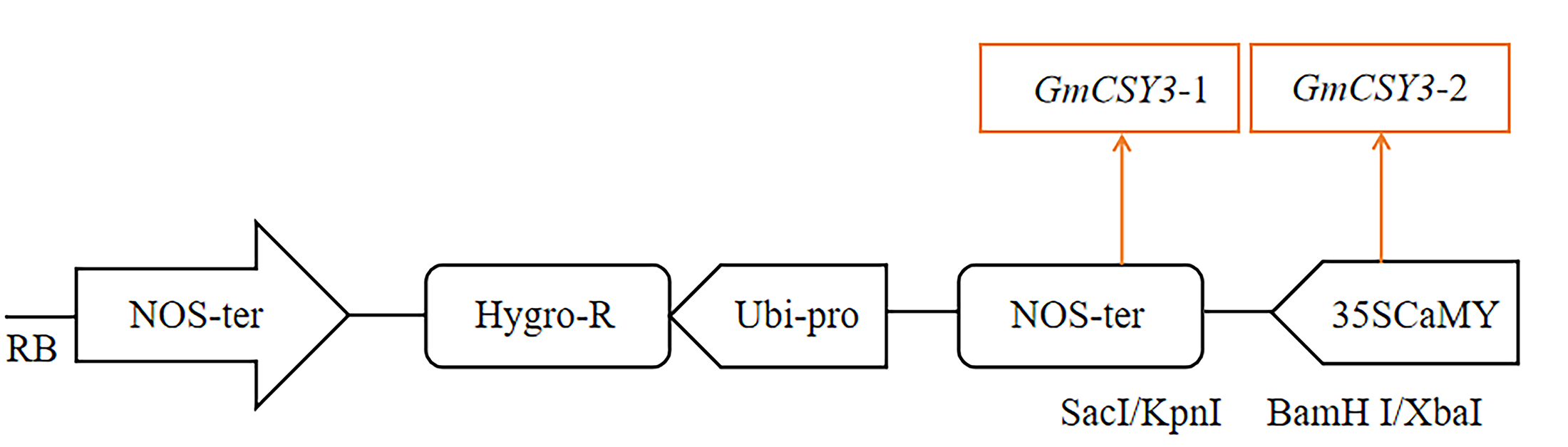

Supplement: Supplementary file 1 [file biology-15-00105-s001.zip › Supplementary Figure S6. Schematic diagram of the construction of RNAi vector pZH01-GmCSY3.tif]

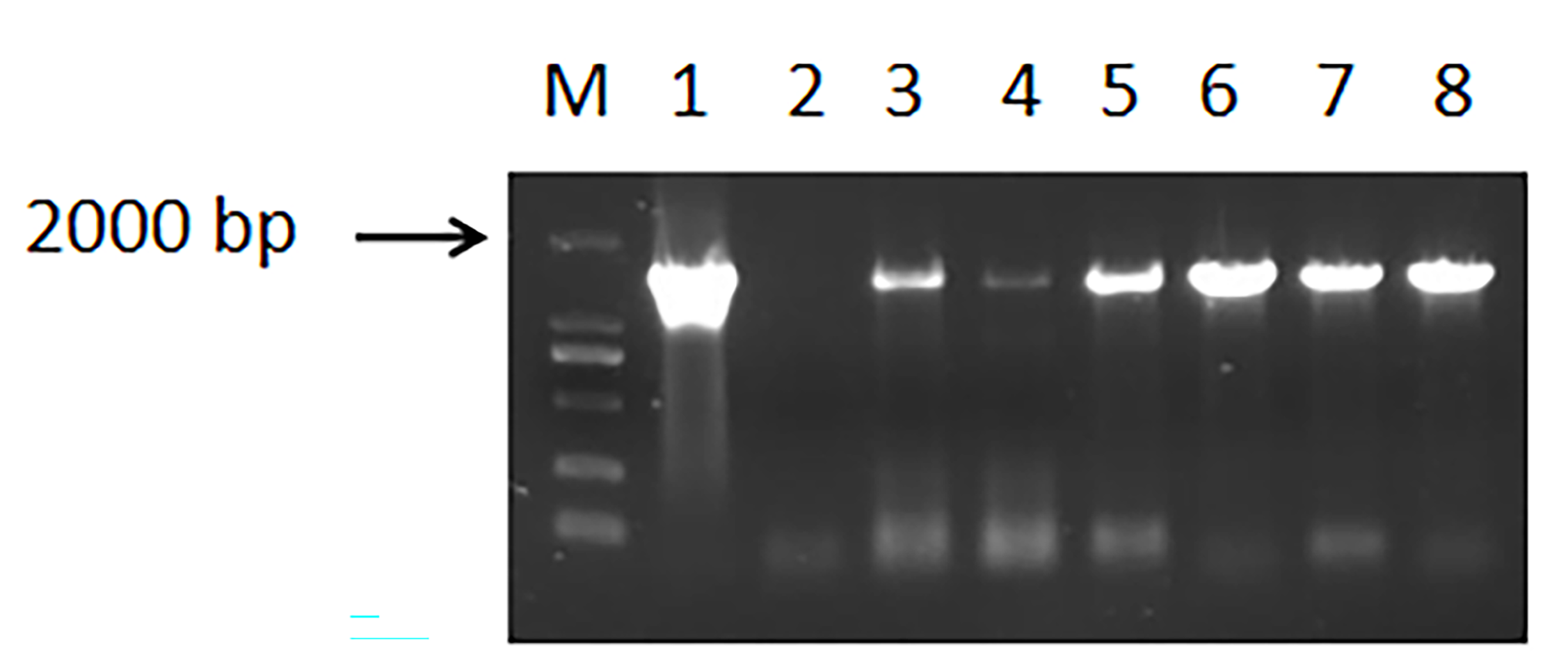

Supplement: Supplementary file 1 [file biology-15-00105-s001.zip › Supplementary Figure S7. Bacterial colony PCR identification of construction of plant expression vector pBI121-GmCSY3.png]

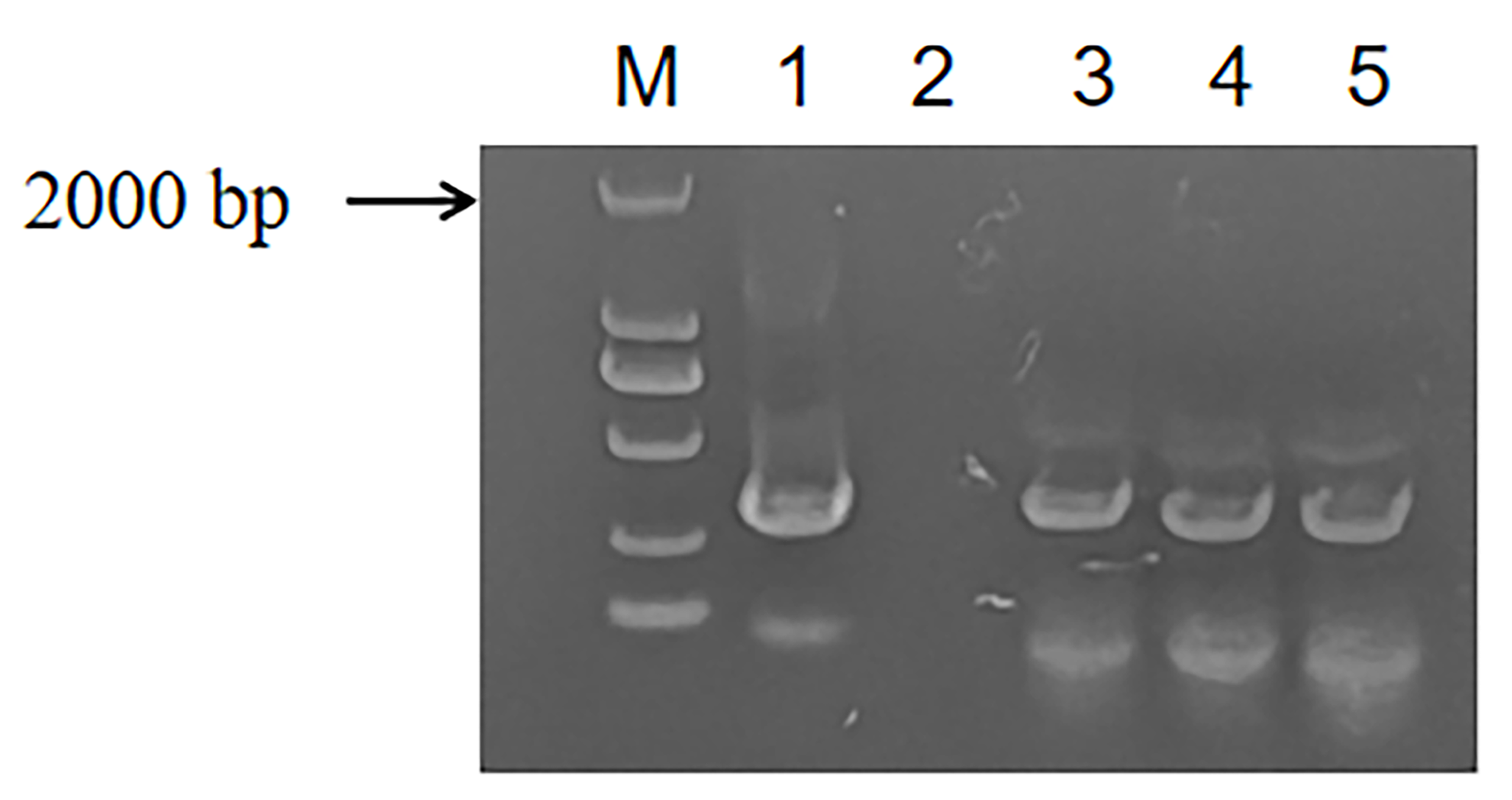

Supplement: Supplementary file 1 [file biology-15-00105-s001.zip › Supplementary Figure S8. Bacterial colony PCR identification of construction of RNAi vector pZH01-GmCSY3.tif]

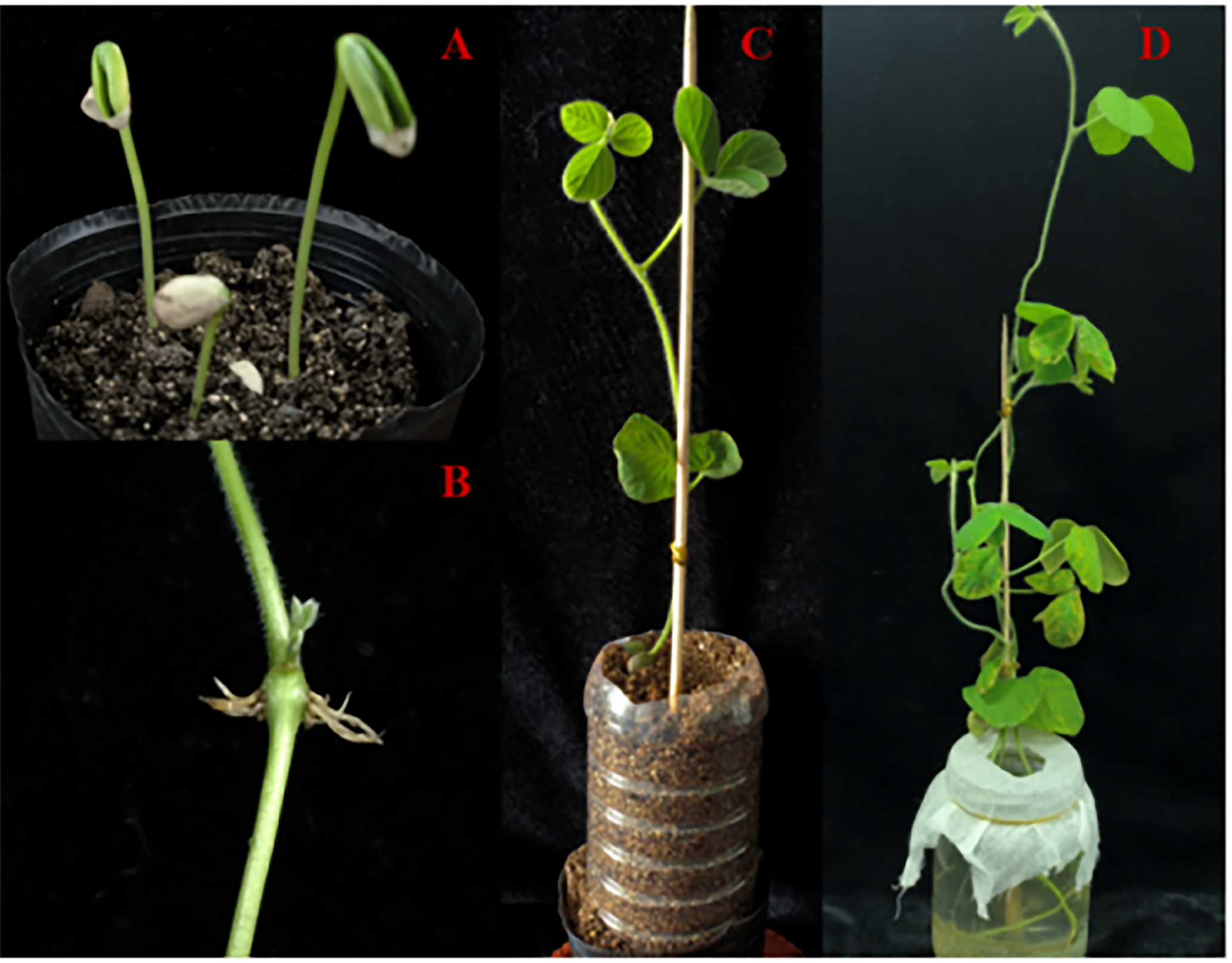

Supplement: Supplementary file 1 [file biology-15-00105-s001.zip › Supplementary Figure S9. Transgenic soybean hairy roots with GmCSY3.tif]
